# Spatial Patterns of High *Aedes aegypti* Oviposition Activity in Northwestern Argentina

**DOI:** 10.1371/journal.pone.0054167

**Published:** 2013-01-17

**Authors:** Elizabet Lilia Estallo, Guillermo Más, Carolina Vergara-Cid, Mario Alberto Lanfri, Francisco Ludueña-Almeida, Carlos Marcelo Scavuzzo, María Virginia Introini, Mario Zaidenberg, Walter Ricardo Almirón

**Affiliations:** 1 Centro de Investigaciones Entomológicas de Córdoba, Instituto de Investigaciones Biológicas y Tecnológicas CONICET-Universidad Nacional de Córdoba, Facultad de Ciencias Exactas, Físicas y Naturales-Ciudad Universitaria, Córdoba, Argentina; 2 INTA EEA San Luis, Villa Mercedes, San Luis, Argentina; 3 Instituto de Virología Dr. JM Vanella, Facultad de Ciencias Médicas, Universidad Nacional de Córdoba, Enfermera Gordillo Gómez s/n, CP X5016GCA Córdoba, Argentina; 4 Instituto de Altos Estudios Espaciales Mario Gulich, Comisión Nacional de Actividades Espaciales (CONAE), Centro Espacial Teófilo Tabanera, Falda del Carmen, Córdoba, Argentina; 5 Ministerio de Salud de la Nación, Argentina; Kenya Medical Research Institute - Wellcome Trust Research Programme, Kenya

## Abstract

**Background:**

In Argentina, dengue has affected mainly the Northern provinces, including Salta. The objective of this study was to analyze the spatial patterns of high *Aedes aegypti* oviposition activity in San Ramón de la Nueva Orán, northwestern Argentina. The location of clusters as hot spot areas should help control programs to identify priority areas and allocate their resources more effectively.

**Methodology:**

Oviposition activity was detected in Orán City (Salta province) using ovitraps, weekly replaced (October 2005–2007). Spatial autocorrelation was measured with Moran’s Index and depicted through cluster maps to identify hot spots. Total egg numbers were spatially interpolated and a classified map with *Ae. aegypti* high oviposition activity areas was performed. Potential breeding and resting (PBR) sites were geo-referenced. A logistic regression analysis of interpolated egg numbers and PBR location was performed to generate a predictive mapping of mosquito oviposition activity.

**Principal Findings:**

Both cluster maps and predictive map were consistent, identifying in central and southern areas of the city high *Ae. aegypti* oviposition activity. A logistic regression model was successfully developed to predict *Ae. aegypti* oviposition activity based on distance to PBR sites, with tire dumps having the strongest association with mosquito oviposition activity. A predictive map reflecting probability of oviposition activity was produced. The predictive map delimitated an area of maximum probability of *Ae. aegypti* oviposition activity in the south of Orán city where tire dumps predominate. The overall fit of the model was acceptable (ROC = 0.77), obtaining 99% of sensitivity and 75.29% of specificity.

**Conclusions:**

Distance to tire dumps is inversely associated with high mosquito activity, allowing us to identify hot spots. These methodologies are useful for prevention, surveillance, and control of tropical vector borne diseases and might assist National Health Ministry to focus resources more effectively.

## Introduction

In 1986, re-infestation of *Aedes aegypti* was detected in northeastern region of Argentina (provinces of Misiones and Formosa) [1], and in a few years it reached even higher levels than before eradication campaign, affecting central and northern region of the country [2]. *Aedes aegypti* might have entered into the northwestern and northeastern provinces from Bolivia, and Paraguay and Brazil, respectively [3]. Early evidence of dengue 2 (DEN-2) virus circulating in northwestern Argentina was reported, with autochthonous cases occurring in Salta Province during 1997 in the localities of Orán (along the National highway N°50), Tartagal, Güemes, and Salvador Mazza (along the National highway N°34), both highways connecting Argentina and Bolivia [2].

Dengue virus circulation was reported only in the northern provinces of Argentina until 2007, when an autochthonous case was detected in Buenos Aires [4]. Between January and June 2009, Argentina suffered its most important dengue outbreak (DEN-1) with more than 26,000 cases, 3 severe dengue cases, and 5 confirmed deaths. Cases affected 14 jurisdictions, 10 of them registering autochthonous cases for the first time (Buenos Aires, Ciudad Autónoma de Buenos Aires, Catamarca, Chaco, Córdoba, Entre Ríos, La Rioja, Santa Fe, Santiago del Estero and Tucumán). This outbreak started in the Orán Department (province of Salta), with the first autochthonous cases being detected during the epidemiologic week (EW) 53 in 2008 and extending until the EW 21 in 2009, when the disease spread south and east, reaching the 35th parallel. Salta was one of the most affected provinces, mainly the northern departments, where the city of San Ramón de la Nueva Orán is located [5].

Introduction and spread of diseases, such as dengue, in a certain region, are strongly influenced by a combination of environmental and anthropic factors, which are heterogeneous throughout geographical space. In the case of dengue, its vector, *Ae. aegypti*, is an urban mosquito, living in and around human dwellings, and also a day-biting mosquito, which feeds preferentially on human blood [6]. Breeding habitats for the mosquito consist of any type of water-holding container, from tree holes or leaves to man-made cisterns, discarded bottles and tires [7]. The National Coordination of Vector Control (Ministry of Health of Argentina) has reported finding larvae in the axils of banana leaves in the province of Misiones [8] and Mangudo *et al*. [9] collected larvae from tree holes in the province of Salta. Studying the distribution of breeding sites within urban areas is a key requirement in assessing dengue transmission risk [10]. Tools for management and processing of spatial data provided by GIS have been gradually integrated in health areas, particularly in the case of transmissible diseases [11], [12]. Geographic information systems (GIS) are analytical tools which allow the study of spatial distribution of vectors, such as mosquitoes [13], [14], and are also helpful in the generation of predictive maps [15], as well as a visual device to examine the spatial location of different control measures and their outcomes [13]. Spatial statistical analysis combined with GIS has led to the quantification and modeling of spatial and temporal correlations in insect populations, resulting in a quantitative analysis of their spatial patterns [16].

GIS technology has an important role in surveillance and control of mosquito-borne diseases. Maps are useful for the identification of spatially and temporally intensified infection areas and potential high-risk populations [17]. Spatial analyzes of entomological and epidemiological variables will be necessary for addressing the challenges of dengue surveillance because they will reveal patterns of dengue virus transmission that can be used to assign progressively more effective intervention strategies [14].

The objective of this study was to analyze the spatial patterns of high *Ae. aegypti* oviposition activity in San Ramón de la Nueva Orán, northwestern Argentina, in relation to different anthropic facilities that might provide suitable breeding grounds. Results should be helpful in control programs to identify priority areas and allocate resources more effectively.

## Materials and Methods

### Study Area

**Figure 1:**
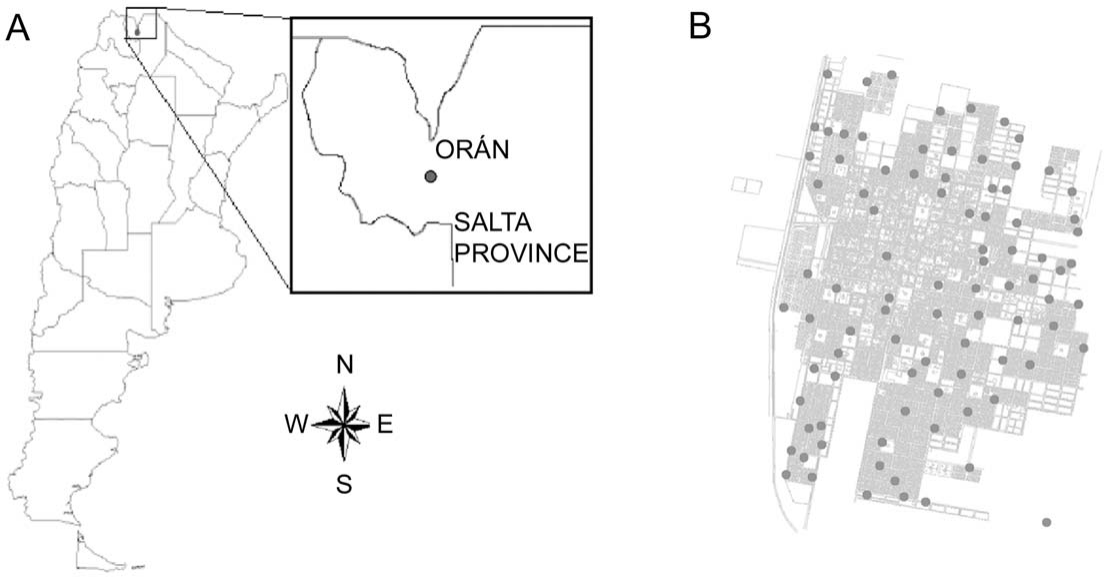
Argentina and San Ramón de la Nueva Orán City. A: Location of the study area, San Ramón de la Nueva Orán, in northwestern Argentina. B: Location of the 90 sampling sites used in the study, in San Ramón de la Nueva Orán (Salta).

San Ramón de la Nueva Orán City (Fig. 1A), hereafter Orán City (23° 08′ S, 64° 20′W) is a town with about 4 by 5 km wide and it is located at a mean altitude of 337 m above sea level in northwestern Argentina. In this region, climate has been classified as subtropical, with summer maximum and minimum temperatures of 44.5°C and 11.5°C, respectively, and winter maximum and minimum temperatures of 38.9°C and −3.6°C. Annual rainfall is 1,000 mm and the annual mean relative humidity is 78%.

### *Aedes aegypti* Areas of High Oviposition Activity

Oviposition activity was detected using ovitraps, a standard tool for monitoring presence of *Ae. aegypti* and therefore their spatial activity [18]. The ovitraps were 350 ml plastic cups which were filled with 250 ml of grass infusion. The infusion was prepared one week before the ovitraps were placed in the field [19]. Sampling was carried out during 2 years, from October 2005 to October 2007, and ovitraps were placed in 90 randomly selected houses (sampling points), distant no longer than 500 m of each other (Fig. 1B). The ovitraps were placed outdoors (gardens or backyards) in private residences, in shaded sites at ground level. We talked with residence owners whom provided permission for the study to be conducted placing the ovitraps in their gardens or backyards. After seven days, each ovitrap was removed and replaced by a new one and eggs in each ovitrap were counted (weekly oviposition). The geographic locations of the 90 sample points were recorded in the field using a global positioning system (GPS) receiver (Garmin GPS MAP 60 CSX). A layer displaying oviposition data was then created with a GIS [20].

Moran’s Index analysis was used to assess the degree of spatial autocorrelation and so determine whether mosquito oviposition activity was randomly distributed or there was significant clustering. The spatial association in *Ae. aegypti* oviposition activity was visualized through cluster maps obtained with Local Indicator of Spatial Association (LISA) [21], which depicted city areas with high oviposition activity. Moran’s Index and LISA cluster maps were calculated using GEODA software [22]. Two LISA cluster maps were first made, for two different periods: November 2005–April 2006 and November 2006–April 2007 (spring and summer time). Then, six LISA cluster maps were made for bimonthly periods: November-December 2005, January-February 2006, March-April 2006, November-December 2006, January-February 2007, and March–April 2007. Eggs numbers were normalized by log transformation (Ln (n+1)).

Moran's *I* ranges from −1 to 1: a value close to 0 indicates spatial randomness while a positive and negative value indicates positive and negative spatial autocorrelation, respectively. Statistical significance was tested using randomization based on 9999 permutations. The weight distance matrix, essential for the computation of spatial autocorrelation statistics, was based on Euclidean distance. The LISA cluster maps are based on P<0.01 (9999 permutations were performed) [21].

### Predictive Mapping of *Aedes aegypti* Oviposition Activity

**Figure 2:**
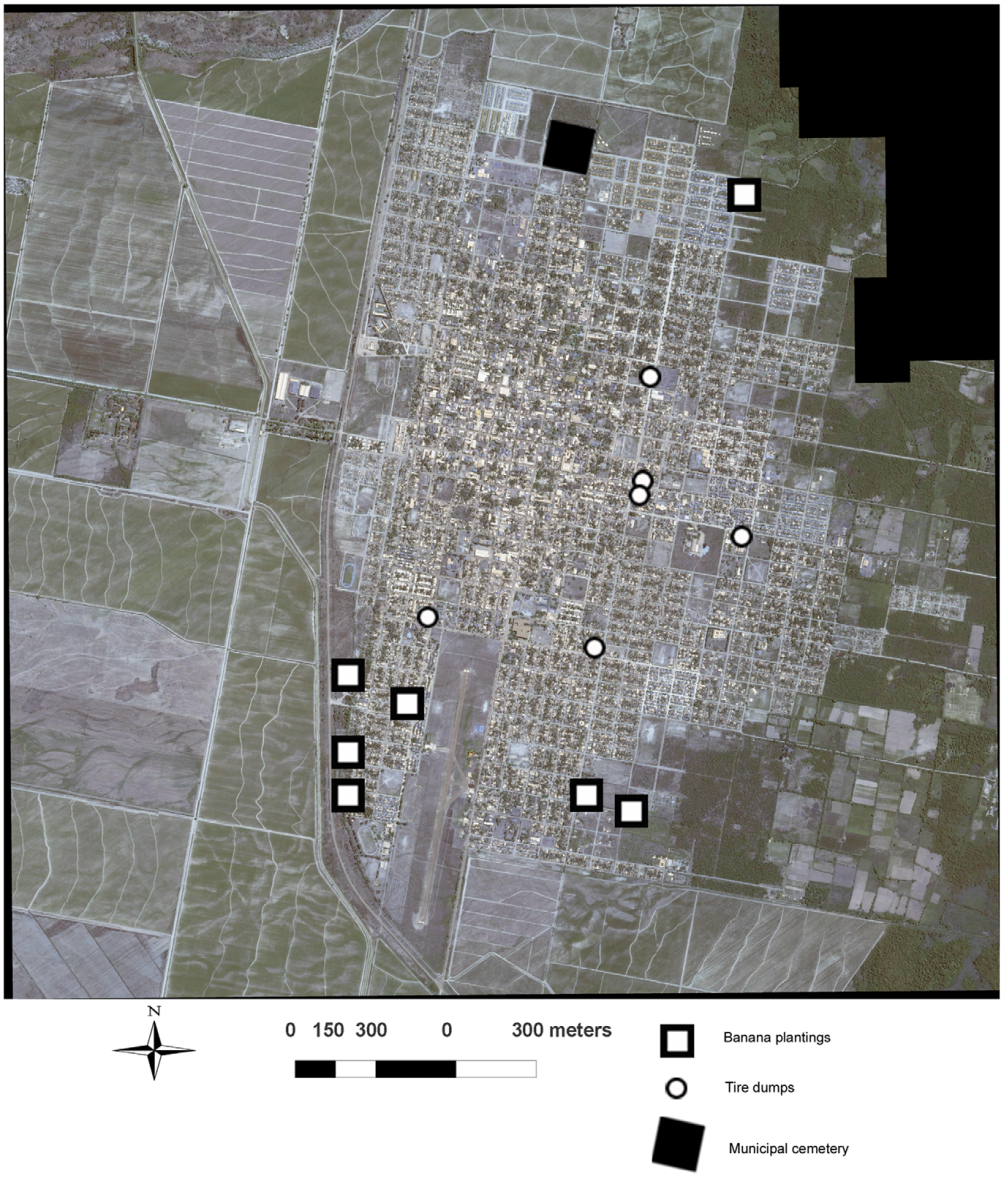
Potential Breading and Resting sites (PBR). Location of the three kinds of PBR sites that included banana plantings, the municipal cemetery and the tire dumps sites in the city.

Many studies have shown that certain habitat types within neighborhoods, can contribute to productivity of *Ae. aegypti* [23]–[25]. In Orán City it is a local habit to accumulate waste on backyards, turning the whole city in a suitable environment for *Ae. aegypti*. Therefore, households are continuously inspected by health authorities. We focused our efforts in identifying others potential breeding and resting (PBR) sites for mosquitoes. We considered three kinds of PBR sites that were located, visited and geo-referenced, recording their positions with a global positioning system (GPS) receiver (Fig. 2). These PBRs included banana plantings, the municipal cemetery and the tire dumps sites in the city. We chose banana plantings because they act as water reservoirs, since water accumulates in the axils of leaves of banana plants, and could allow mosquitoes breeding. We considered the municipal cemetery after visiting it and observing many discarded bottles and containers that could accumulate rain water. Finally we chose the tire dumps because tires are stored outdoors, accumulating rain water, where we saw mosquitoes eggs and larvae.

In order to determine whether PBR sites are associated to *Ae. aegypti* oviposition abundance, a logistic regression model was developed using GIS Idrisi ANDES [26] and InfoStat software [27]. In this study, high/low *Ae. aegypti* oviposition abundance was used instead of presence/absence as the response variable, since *Ae. aegypti* is widespread in Orán city and individuals were present in all ovitraps. Total numbers of eggs counted for the whole period of study (October 2005–October 2007) were spatially interpolated fitting a linear model to local neighborhood of data with ordinary least squares to produce a map of oviposition abundance. The resulting image was classified in two classes (low vector activity: less than 3201 eggs and high vector activity: more than 3021 eggs) according to the median number of eggs found in ovitraps. For each PBR site, the Euclidean distance to the nearest source cell was generated. Distances to PBR sites were the predictive variables in our model of oviposition activity. All datasets were exported to InfoStat software in order to run the analysis. A stepwise logistic regression analysis (p<0.05) was performed to develop the best model. A predictive map reflecting probability of oviposition activity was produced.

The logistic model success was evaluated by the receiver operating characteristic (ROC). The specificity (percentage of correctly classified low abundance traps) and sensitivity (percent of correctly classified high abundance traps) were also calculated to judge the overall fit of the model.

## Results

### *Aedes aegypti* Areas of High Oviposition Activity

**Figure 3:**
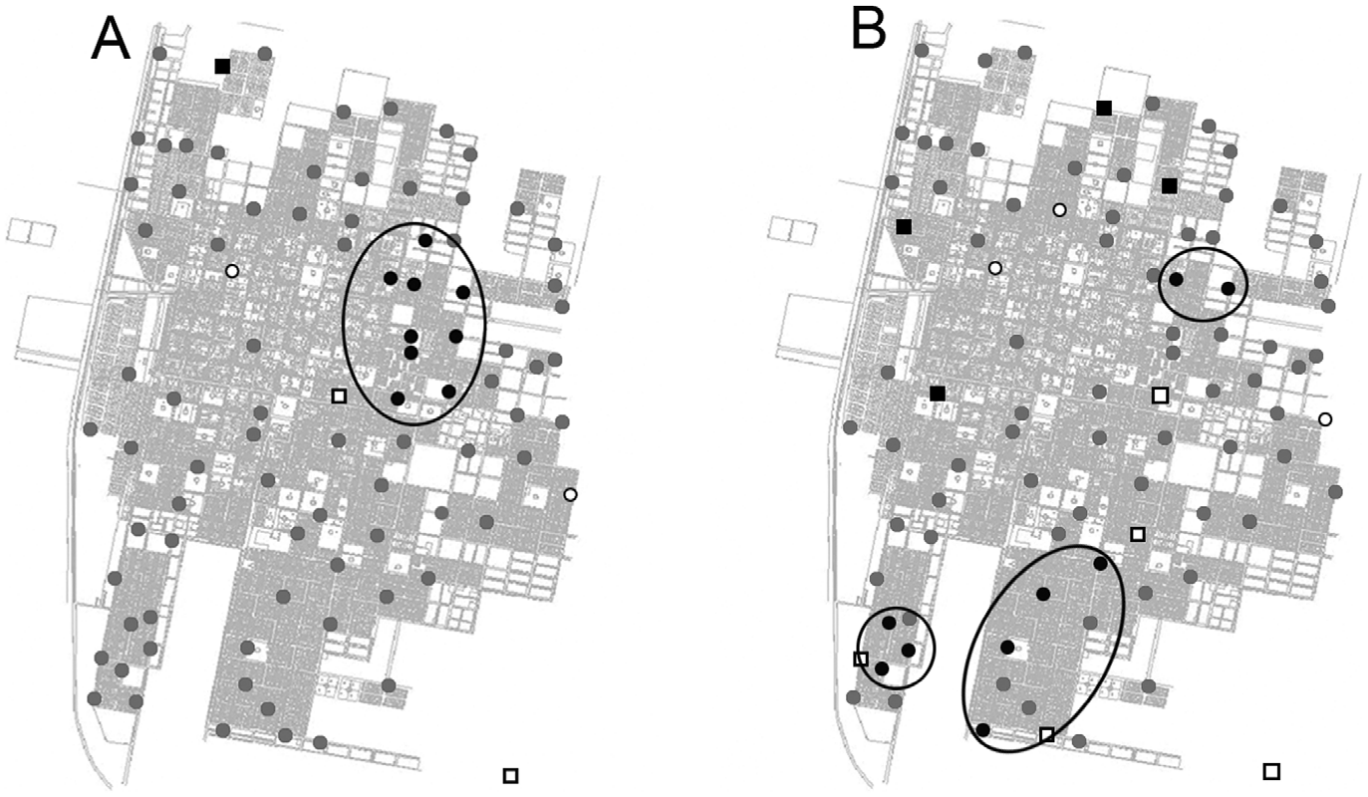
Lisa cluster maps. A: November 2005 to April 2006 period. B: November 2006 to April 2007 period. The black circle and black square locations are indications of spatial clusters (respectively, high surround by high, and low surrounded by low values). The white circle and white square are indications of spatial outliers (respectively, high surrounded by low, and low surrounded by high values). The maps are based on P<0.01 (after 9999 permutations).

Eggs collected in ovitraps peaked between January and February. Positive spatial autocorrelation in egg number was found (Table 1); the Moran’s Index showed significant spatial clustering in oviposition activity (P<0.05). LISA cluster maps depict the significant spatial clustering and classify those spatial clusters by type of association (high-high, high-low, low-low, low-high). For the complete sampling period (November 2005 to April 2006 and November 2006 to April 2007) cluster maps clearly indicated a high-high spatial clustering on the central (Fig. 3A) and southern areas of Orán City (Fig. 3B).

**Table 1:** Moran’s Index values (P<0.01) for the period of 6 month between November and April of each sampling year, and for the bimonthly of January-February when oviposition is higher.

	Moran’s *I*
	500 m ( 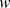 )
NOV05–APR06	0.1293
NOV06–APR07	0.1420
JAN–FEB 2006	0.1761
JAN–FEB 2007	0.0901

Weigh distance 500 meters.

**Figure 4:**
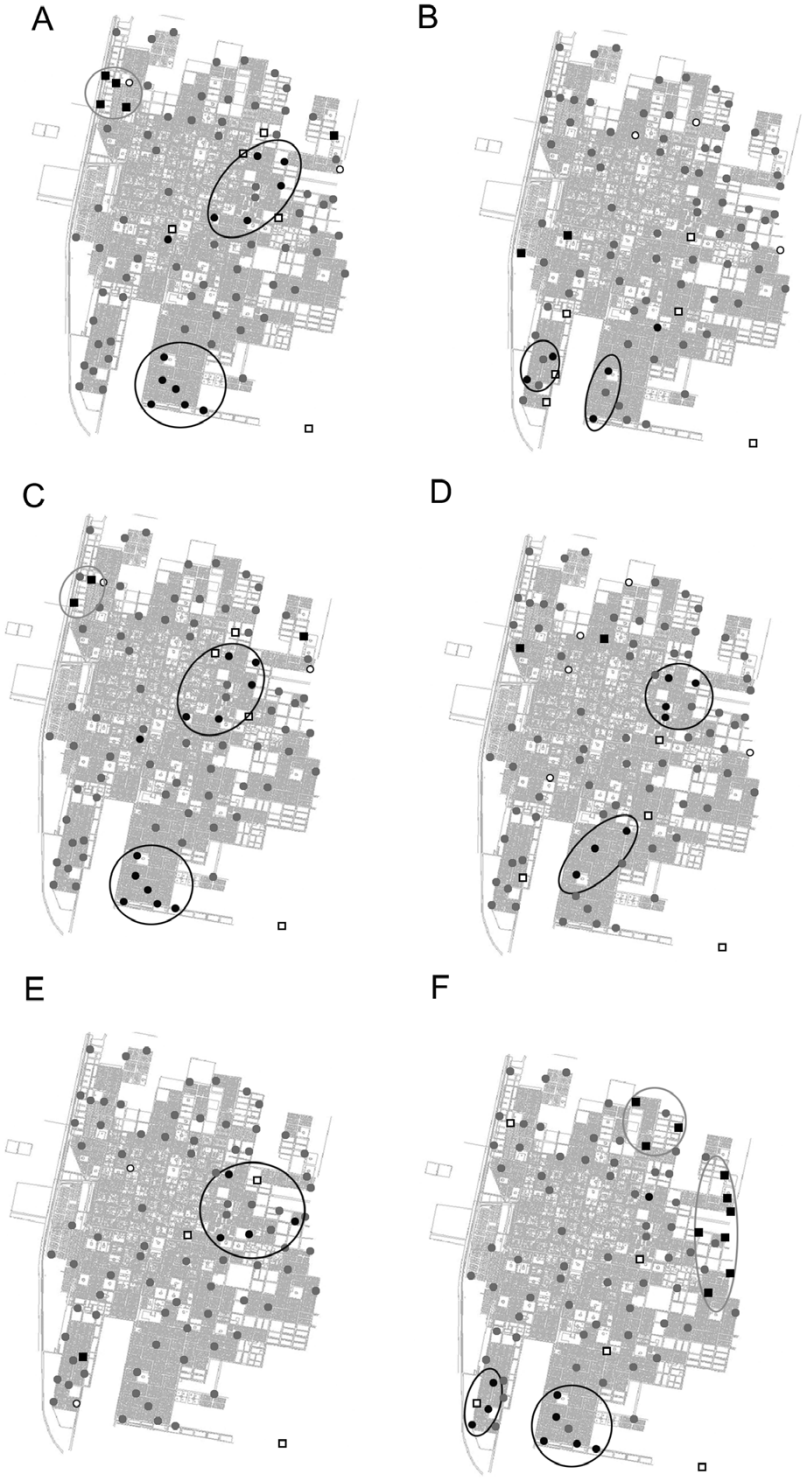
Lisa clusters maps for bimonthly periods. November-December, 2005 (A), 2006 (B); January-February, 2006 (C), 2007 (D); March-April, 2006 (E), 2007 (F). The black circle and black square locations are indications of spatial clusters (respectively, high surround by high, and low surrounded by low values). The white circle and white square are indications of spatial outliers (respectively, high surrounded by low, and low surrounded by high values). The maps are based on P<0.01 (after 9999 permutations).

The bimestrial LISA cluster maps (Fig. 4) showed the spatial and temporal variation in oviposition activity. Oviposition activity was higher during January-February (Fig. 4C–D), with a high-high spatial cluster on central and southern areas of Orán on both consecutive sampling years.

### Predictive Mapping of *Aedes aegypti* Oviposition Activity

**Figure 5:**
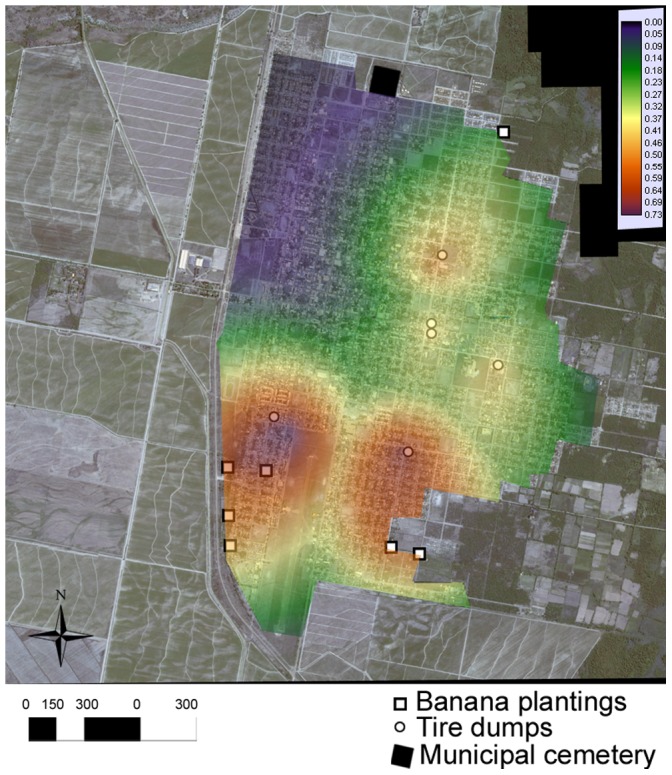
Predictive map. The map reflecting probability of oviposition activity delimitated an area of maximum probability of *Ae. aegypti* oviposition activity in the south of Orán city.

Each kind of PBR site has a unique spatial pattern. Banana plantings are in the northern and southern perimeter of the city. The cemetery is also in a peripheral location, north of the city (Fig. 2). The tire dumps sites; however, are located in the center and south of the city (Fig. 2). A logistic regression model was successfully developed to predict *Ae. aegypti* oviposition activity based on distance to PBR sites (Fig. 5). Distance to dump sites and to banana plantings were retained in the model (Table 2), with tire dumps having the strongest association with mosquito oviposition activity. The distance to the cemetery was excluded from the final model due to be deficient in predicting mosquito abundance. The predictive map (Fig. 5) delimitated areas of maximum probability of abundance near the tire dumps and banana plantings located at central and southern areas of Orán City (Fig. 5). The overall fit of the model was acceptable (ROC = 0.77), obtaining 99% of sensitivity and 75.29% of specificity.

**Table 2:** Final logistic regression model for *Aedes Aegypti* abundance prediction.

Variable	Coefficient	Wald statistic	p
Distance to tire dumps	−1.1×10^−3^	4.78	0.0289
Distance to banana plantings	−4.2×10^−4^	1.03	0.3109

## Discussion

A spatial analysis was performed to determine the city areas with high *Ae. aegypti* oviposition activity in Orán City through the use of GIS techniques, calculating the Moran’s Index for spatial autocorrelation and developing LISA clusters maps, which showed high spatial clustering in *Ae. aegypti* oviposition activity or hot spots in the center and southern areas of Orán City.

Moran’s Index analysis has been widely used in the study of insect patterns. Ryan *et al*. [28]used this index to determine whether adult mosquitoes of 4 different species (*Ochlerotatus vigilax*, *Coquillettidia linealis*, *Culex annulirotris* and *Ochlerotatus notoscriptus*) in Queensland, Australia, were randomly distributed, or there was significant clustering of traps with high or low number of mosquitoes, focusing in the delimitation of geographic areas with consistently high or low number of mosquitoes. This allows control activities to be focused in areas with high levels of arbovirus transmission. In this study, a positive spatial autocorrelation was found, indicating aggregation in egg numbers in the central and southern areas of Orán City. Control activities should be focused in those areas, in order to attain a more efficient use of resources in control programs. We observed persistence of clusters or hot spots for two periods of peak activity (January-February 2005–2006 and 2006–2007) (Fig. 4C–D) at the same location (aggregation of egg number in the center and southern areas of Orán City) indicating spatial stability. This finding is important for operational vector control purposes.

Barrera [29] showed through temporal analyses in two neighborhoods of Puerto Rico, a relatively high concordance in the rank order of adult *Ae. aegypti* trap productivity in time, which translates into a pattern of spatial stability of *Ae. aegypti* females in both neighborhoods. Spatial stability has also been reported for tsetse flies in Luke community, Ethiopia [30].

According to Moore et al. [31], the abundance of *Ae. aegypti* is proportional to the availability of potential breeding sites. Tinker [32] found a positive association between the infestation rates and density of containers. We built a predictive map with distances to potential breeding and resting sites, where tire dump sites resulted significant predictors. Tire dump sites were located mostly on the southern city areas, in consistency with high oviposition seen in cluster maps. Stein *et al*. [24] reported that car tires were one of the preferred kinds of containers used by *Ae. aegypti* to lay eggs in Chaco province, Argentina (northeastern). Since the National Coordination of Vector Control (Ministry of Health of Argentina) has reported finding larvae in the axils of banana leaves in the province of Misiones [8] and *Ae. aegypti* larvae have been detected in banana leaf axils in Cuba [33], banana plantings were also considered as potential breeding sites in this study. Through the use of GIS technologies we established a spatial relationship between availability of these kinds of breeding sites and *Ae. aegypti* oviposition activity, which was concentrated in the center and south of Orán City. Both banana plantings and tire dumps sites in Orán city would be important in *Ae. aegypti* spatial dynamics, although *Ae. aegypti* productivity from natural containers (as banana plantings) is usually low compared to artificial containers like used tires [34], as have also been observed in our study.

According to Brooker et al. [35], who used spatial analysis to study epidemiology of *Plasmodium vivax* in Afghanistan, GIS helped to highlight areas of disease risk and could clarify ecologic risk factors for disease transmission. Also, maps for cutaneous Leishmaniasis in Colombia showed a significant impact in control planning, as they identify geographic areas with consistently high or low density of vectors and allow control activities to be focused in areas with the greatest risk of transmission [36].

In consistency with our results, during the 2009 outbreak in Orán, the first cases of dengue occurred in the south of the city and, spread then to the centre and east (Palacios et al. unpublished data), where the potential breeding and resting sites were identified, pointed as areas of high density of adult *Ae. aegypti* and elevated risk of acquiring dengue. Our predictive map, based on distances to PBR sites, showed the southern area of the city to be the one with more probability of high mosquito activity. We emphasize that location of these areas, as well as the methodology applied to identify them, constitute a powerful aid to National Health Ministry in the task of preventing and/or controlling mosquitoes and future dengue outbreaks in the city.
